# Serum Uric Acid Is Positively Associated with Handgrip Strength among Japanese Community-Dwelling Elderly Women

**DOI:** 10.1371/journal.pone.0151044

**Published:** 2016-04-14

**Authors:** Ryuichi Kawamoto, Daisuke Ninomiya, Yoshihisa Kasai, Tomo Kusunoki, Nobuyuki Ohtsuka, Teru Kumagi, Masanori Abe

**Affiliations:** 1 Department of Community Medicine, Ehime University Graduate School of Medicine, Ehime, 791–0295, Japan; 2 Department of Internal Medicine, Seiyo Municipal Nomura Hospital, Ehime, 797–1212, Japan; IPK, GERMANY

## Abstract

Serum uric acid (UA) has strong anti-oxidant properties. Muscle strength and mass decrease with age, and recently, this decrease has been defined as sarcopenia. Sarcopenia may be triggered by oxidative stress. We investigated whether serum UA is associated with handgrip strength (HGS), which is a useful indicator of sarcopenia, among Japanese community-dwelling elderly persons. The present study included 602 men aged 72 ± 7 years and 847 women aged 71 ± 6 years from a rural village. We examined the cross-sectional relationship between serum UA and HGS. In both genders, HGS increased significantly with increased serum UA levels. A multiple linear regression analysis using HGS as an objective variable and various confounding factors as explanatory variables showed that in men age, drinking status, high-density lipoprotein cholesterol (HDL-C), low-density lipoprotein cholesterol (LDL-C), and estimated glomerular filtration ratio (eGFR_CKDEPI_) were independently and significantly associated with HGS, and in women, serum UA as well as age, body mass index, drinking status, diastolic blood pressure, and eGFR_CKDEPI_ were independently and significantly associated with HGS. In women, age and multivariate-adjusted HGS were significantly higher in the Quartile-3 (4.8–5.4 mg/dL) and Quartile-4 groups (5.5–9.3 mg/dL) of serum UA than in the lower groups (0.7–4.7 mg/dL). These results suggest that serum UA may have a protective role in aging-associated decline in muscle strength in community-dwelling elderly women.

## Introduction

Serum uric acid (UA) in humans is the end-product of purine metabolism, and a number of studies have shown that hyperuricemia is an important risk factor for systemic inflammation [1], endothelial dysfunction [2], hypertension [3], impaired fasting glucose [4], cardiovascular disease (CVD) and CVD mortality [5]. Despite a strong association between serum UA level and various CVDs in humans, UA is not considered as having a pathogenetic role in these conditions, and instead, is considered to be a reactive oxygen species (ROS) scavenger, and to have strong anti-oxidant properties [6, 7].

On the other hand, skeletal muscle strength and mass decrease with age, and recently, this clinical condition, which is defined as an important component for the evaluation of sarcopenia, has been characterized by progressive loss of motor units and wasting of muscle fibers resulting in decreased muscle function [8, 9]. The molecular mechanisms leading to sarcopenia have not been completely understood; but increased oxidative damage in muscle cells that accumulates throughout one's lifetime represents one of the most accepted underlying pathways [10, 11]. Thus, increased UA may play an importantly protective role in counteracting the excessive production of free radicals, especially in elder persons, who are increasingly becoming a large portion of the population. Macchi et al. [12, 13] demonstrated that higher circulating levels of serum UA are prospectively associated with higher handgrip strength (HGS) in middle aged and older persons. Unfortunately, there are few studies showing the protective effect of serum UA on health status and aging phenotypes.

We hypothesized that serum UA is associated with muscle strength. To confirm this hypothesis, we investigated whether serum UA is associated with HGS, which is a useful indicator of sarcopenia [14], among Japanese community-dwelling elderly persons.

## Materials and Methods

### Subjects

The present study was designed as part of the Nomura study [15, 16]. The study population aged ≥60 years was selected through a community-based annual check-up process from the Nomura health and welfare center in a rural town located in Ehime prefecture, Japan. The physical activity level of subjects (e.g., exercise habits), information on medical history, present conditions, and medications (e.g., antihypertensive, antidyslipidemic, antidiabetic, and uric acid lowering medication) were obtained by interview using a structured questionnaire. For all these individuals, overnight fasting plasma samples were made available. Participants with an estimated glomerular filtration ratio (eGFR) of <30 ml/min/1.73 m^2^ and uric acid lowering medication that could affect uric acid were excluded. The study complies with the Declaration of Helsinki, and was approved by the ethics committee of Ehime University School of Medicine with written informed consent obtained from each subject.

### Evaluation of Risk Factors

Information on demographic characteristics and risk factors was collected using clinical files. Body mass index (BMI) was calculated by dividing weight (in kilograms) by the square of the height (in meters). Smoking status was defined as the number of cigarette packs per day multiplied by the number of years smoked (pack・year), and the participants were classified into never smokers, past smokers, light smokers (<20 pack year) and heavy smokers (≥20 pack year). Daily alcohol consumption was measured using the Japanese liquor unit in which a unit corresponds to 22.9 g of ethanol, and the participants were classified into never drinkers, occasional drinkers (<1 unit/day), and daily drinkers (≥1 unit/day). We measured systolic blood pressure (SBP) and diastolic blood pressure (DBP) in the right upper arm of participants in the sedentary position using an automatic oscillometric blood pressure recorder while the subjects were seated after having rested for at least 5 min. Appropriate cuff bladder size was determined at each visit based on arm circumference. Triglycerides (TG), high-density lipoprotein cholesterol (HDL-C), low-density lipoprotein cholesterol (LDL-C), fasting plasma glucose (FPG), creatinine (Cr), and serum UA were measured during fasting. eGFR was calculated using CKD-EPI equations modified by a Japanese coefficient (eGFR_CKDEPI_): Male, Cr ≤0.9 mg/dl, 141 × (Cr/0.9) –^0.411^ × 0.993 ^age^ × 0.813; Cr >0.9 mg/dl, 141 × (Cr/0.9) –^1.209^ × 0.993 ^age^ × 0.813; Female, Cr ≤0.7 mg/dl, 144 × (Cr/0.7) –^0.329^ × 0.993 ^age^ × 0.813; Cr >0.7 mg/dl, 144 × (Cr/0.7) –^1.209^ × 0.993 ^age^ × 0.813 [16]. Chronic kidney disease (CKD) was defined as an eGFR_CKDEPI_ of <60 ml/min/1.73 m^2^.

### HGS test

The subject holds the dynamometer in the hand to be tested, with the arm at right angles and the elbow by the side of the body. The handle of the dynamometer is adjusted if required—the base should rest on the first metacarpal (heel of palm), while the handle should rest on the four middle fingers. When ready the subject squeezes the dynamometer with maximum isometric effort, which is maintained for about 5 seconds. No other body movement is allowed [17]. The mean of two right and left measurements was used for analysis.

### Statistical Analysis

Statistical analysis was performed using IBM SPSS Statistics Version 20 (Statistical Package for Social Science Japan, Inc., Tokyo, Japan). All valuesare expressed as mean ± standard deviation (SD), unless otherwise specified. Data for TG was skewed, and log-transformed for analysis. Subjects were divided into four groups based on quartile of serum UA according to gender, and differences among the groups were analyzed by ANOVA for the continuous variables or the χ^2^ -test for the categorical variables. Multiple linear regression analysis was used to evaluate the contribution of each confounding factor for HGS. ANCOVA was performed using a general linear model approach to determine the association between the confounding factors and HGS. In these analyses, HGS was the dependent variable, the four categories of serum UA were the fixed factors, and all confounding factors in the multiple linear regression analysis were added as covariates. The interactive effect of gender and serum UA levels on HGS was evaluated using a general linear model. A *p*-value <0.05 was considered significant.

## Results

### Characteristics of subjects categorized by gender

Gender-specific characteristics of the subjects are illustrated in Table 1. The study included 602 men aged 72 ± 7 (range, 60–95) years and 847 women aged 71 ± 6 (range, 60–90) years. HGS was 32.5 ± 7.2 kg in men and 21.1 ± 3.9 kg in women, and serum UA was 5.9 ± 1.3 in men and 4.7 ± 1.1 in women. The prevalence of physical activity was 65.6% in men and 64.9% in women (*p* = 0.780). In men, BMI, smoking status, drinking status, DBP, TG, HbA1c, prevalence of antidiabetic medication, prevalence of CKD, serum UA, and HGS were significantly higher in men, but HDL-C, LDL-C, prevalence of antidyslipidemic medication, and eGFR_CKDEPI_ were significantly lower. There were no gender differences regarding age, exercise habits, SBP, and prevalence of antihypertensive medication.

**Table 1:** Characteristics of subjects by gender.

	Men	Women	
Characteristics N = 1,449	N = 602	N = 847	*P* -value*
Age (years)	72 ± 7	71 ± 6	0.095
Body mass index (kg/m^2^)	23.0 ± 2.9	22.6 ± 3.2	0.006
Exercise habits (No = 0, Yes = 1) (%)	38.4	39.4	0.702
Smoking status† (never/past/light/heavy (%))	43.4/39.4/2.2/15.1	97.0/1.9/0.4/0.7	<0.001
Drinking Status‡ (never/occasional/ daily (%))	25.2/23.1/51.7	72.3/22.1/5.7	<0.001
Systolic blood pressure (mmHg)	137 ± 17	137 ± 17	0.606
Diastolic blood pressure (mmHg)	79 ± 10	77 ± 9	<0.001
Antihypertensive medication (No = 0, Yes = 1) (%)	47.0	46.5	0.873
Triglycerides (mg/dl)	88 (66–129)	87 (66–117)	0.015
HDL cholesterol (mg/dl)	62 ± 16	68 ± 16	<0.001
LDL cholesterol (mg/dl)	113 ± 29	125 ± 29	<0.001
Antidyslipidemic medication (No = 0, Yes = 1) (%)	13.0	30.9	<0.001
Hemoglobin A 1c (%)	5.9 ± 0.8	5.8 ± 0.5	0.014
Antidiabetic medication (No = 0, Yes = 1) (%)	14.5	5.7	<0.001
eGFR_CKDEPI_ (ml/min/1.73 m^2^)	69.7 ± 10.5	71.7 ± 9.2	<0.001
Chronic kidney disease# (No = 0, Yes = 1) (%)	16.3	10.5	0.001
Serum uric acid (mg/dL)	5.9 ± 1.3	4.7 ± 1.1	<0.001
Handgrip strength (kg)	32.5 ± 7.2	21.1 ± 3.9	<0.001

HDL, high-density lipoprotein; LDL, low-density lipoprotein. Data presented are mean ± standard deviation. Data for triglycerides is skewed, and is presented as median (interquartile range) values.

† Smoking status was defined as the number of cigarette packs per day multiplied by the number of years smoked (pack・year), and the participants were classified into never smokers, past smokers, light smokers (<20 pack year) and heavy smokers (≥20 pack year).

‡ Daily alcohol consumption was measured using the Japanese liquor unit in which a unit corresponds to 22.9 g of ethanol, and the participants were classified into never drinkers, occasional drinkers (<1 unit/day), daily drinkers (≥1 unit/day).

# Chronic kidney disease was defined as an glomerular filtration ratio _CKDEPI_ of <60 ml/min/1.73 m^2^.

* *P*-value: student’s test or χ^2^ test.

#### Characteristics of subjects categorized by gender and quartiles of serum UA

We thought that sex-specific analyses were also required because serum UA level and handgrip strength are higher in men than in women. Gender-specific characteristics of the subjects categorized by gender and quartiles of serum UA level are illustrated in Table 2 and Table 3. In men, BMI, TG, and prevalence of CKD were significantly higher with increased serum UA, but age, HDL-C, HbA1c levels, and eGFR_CKDEPI_ were significantly lower (Table 2). In women, BMI, presence of antihypertensive medication, TG, HbA1c, and prevalence of CKD were significantly higher, but HDL-C levels and eGFR_CKDEPI_ were significantly lower with increased serum UA (Table 3).

**Table 2:** Characteristics of male subjects categorized by quartiles of serum uric acid.

Men	Serum uric acid (mg/dL),				
	Quartile-1	Quartile-2	Quartile-3	Quartile-4	
	
	0.6–5.1	5.2–5.8	5.9–6.7	.8–9.4	
Characteristics N = 602	N = 165	N = 136	N = 16	N = 140	*P* for trend*
Age (years)	74 ± 8	71 ± 7	71 ± 7	72 ± 7	0.002
Body mass index (kg/m^2^)	22.5 ± 2.8	23.1 ± 2.9	23.1 ± 3.1	23.5 ± 2.9	0.017
Exercise habits, N (%)	41.8	35.3	36.6	39.3	0.654
Smoking status (never/past/light/heavy (%))	43.6/42.4/1.8/12.1	45.6/30.9/4.4/19.1	43.5/37.9/1.2/17.4	40.7/45.7/1.4/12.1	0.170
Drinking Status (never/occasional/ light/heavy (%))	30.3/21.2/48.5	25.0/25.0/50.0	26.7/24.8/48.4	17.9/21.4/60.7	0.196
Systolic blood pressure (mmHg)	139 ± 17	136 ± 17	134 ± 17	137 ± 15	0.069
Diastolic blood pressure (mmHg)	79 ± 11	78 ± 9	79 ± 9	81 ± 10	0.053
Antihypertensive medication (%)	47.9	40.4	44.1	55.7	0.065
Triglycerides (mg/dl)	83 (63–119)	83 (66–117)	90 (69–140)	99 (73–149)	0.001
HDL cholesterol (mg/dl)	65 ± 19	61 ± 16	60 ± 15	60 ± 16	0.039
LDL cholesterol (mg/dl)	111 ± 28	115 ± 28	115 ± 30	112 ± 30	0.472
Antidyslipidemic medication (%)	12.7	13.2	13.7	12.1	0.982
Hemoglobin A 1c (%)	6.1 ± 1.3	5.7 ± 0.5	5.8 ± 0.6	5.8 ± 0.5	0.001
Antidiabetic medication (%)	18.8	12.5	11.2	15.0	0.225
eGFR_CKDEPI_ (ml/min/1.73 m^2^)	72.2 ± 8.9	71.7 ± 10.0	69.4 ± 8.8	65.1 ± 12.7	<0.001
Chronic kidney disease (%)	8.5	14.7	14.9	28.6	<0.001

Data presented are mean ± standard deviation. Data for triglycerides is skewed, and is presented as median (interquartile range) values.

* *P* for trend: Kruskal Wallis test or χ^2^ test.

**Table 3:** Characteristics of female subjects categorized by quartiles of serum uric acid.

Women	Serum uric acid (mg/dL)				
	Quartile-1	Quartile-2	Quartile-3	Quartile-4	
	0.7–4.0	4.1–4.7	4.8–5.4	5.5–9.3	
Characteristics N = 847	N = 233	N = 222	N = 198	N = 194	*P* for trend*
Age (years)	71 ± 7	71 ± 6	71 ± 7	73 ± 6	0.060
Body mass index (kg/m^2^)	21.7 ± 3.0	22.2 ± 2.9	23.2 ± 3.2	23.6 ± 3.3	<0.001
Exercise habits (%)	35.6	43.7	37.4	41.2	0.293
Smoking status (never/past/light/heavy (%))	97.9/1.3/0/0.9	98.2/1.4/0/0.5	96.0/2.5/1.0/0.5	95.9/2.6/0.5/1.0	0.675
Drinking Status (never/occasional/ light/heavy (%))	73.4/21.9/4.7	77.9/17.1/5.0	70.2/25.3/4.5	66.5/24.7/8.8	0.111
Systolic blood pressure (mmHg)	137 ± 18	136 ± 17	138 ± 17	139 ± 16	0.387
Diastolic blood pressure (mmHg)	77 ± 10	76 ± 9	77 ± 9	77 ± 9	0.515
Antihypertensive medication (%)	36.9	40.1	53.0	58.8	<0.001
Triglycerides (mg/dl)	82 (63–108)	81 (64–109)	87 (65–118)	97 (73–138)	<0.001
HDL cholesterol (mg/dl)	68 ± 16	71 ± 17	68 ± 15	64 ± 16	<0.001
LDL cholesterol (mg/dl)	125 ± 29	124 ± 29	126 ± 28	124 ± 32	0.943
Antidyslipidemic medication (%)	24.9	30.6	32.3	37.1	0.054
Hemoglobin A 1c (%)	5.7 ± 0.4	5.8 ± 0.5	5.7 ± 0.4	5.9 ± 0.5	<0.001
Antidiabetic medication (%)	3.4	7.7	4.0	7.7	0.096
eGFR_CKDEPI_	75.7 ± 7.0	73.3 ± 7.7	70.5 ± 8.4	66.2 ± 11.0	<0.001
Chronic kidney disease (%)	2.1	6.3	11.1	24.7	<0.001

Data presented are mean ± standard deviation. Data for triglycerides is skewed, and is presented as median (interquartile range) values.

* *P* for trend: Kruskal Wallis test or χ^2^ test.

#### HGS of subjects categorized by gender and quartiles of serum UA

As shown in Table 4, HGS increase significantly with increased serum UA levels in both genders, and in men HGS values in Quartile-2, Quartile-3, and Quartile-4 were significantly greater than those in Quartile-1, and in women HGS value in Quartile-3 and Quartile-4 were significantly greater than those in Quartile-1.

**Table 4:** Handgrip strength of subjects categorized by gender and quartile of serum uric acid.

	Serum uric acid (mg/dL)				
	Quartile-1	Quartile-2	Quartile-3	Quartile-4	
	0.6–5.1	5.2–5.8	5.9–6.7	6.8–9.4	
**Men N = 602**	**N = 165**	**N = 136**	**N = 161**	**N = 140**	*P* for trend*
Mean handgrip strength (kg)	30.5 (29.4–31.7)	33.0 (31.8–34.2)†	33.6 (32.5–34.7) ‡	33.1 (31.9–34.2) †	<0.001
	**0.7–4.0**	**4.1–4.7**	**4.8–5.4**	**5.5–9.3**	
**Women N = 847**	**N = 233**	**N = 222**	**N = 198**	**N = 194**	*P* for trend*
Mean handgrip strength (kg)	20.4 (19.9–20.9)	20.6 (20.1–21.1)	22.2 (21.6–22.7) ‡ #	21.4 (20.8–22.0) †	<0.001

Mean handgrip strength was obtained by averaging the right and left handgrip strengths. Data presented was mean (95% confidence interval).

* *P* for trend: Kruskal Wallis test.

† *P*<0.05

‡ *P* = 0.001 vs. Quartile-1; and

# *P*<0.001 vs. Quartile-2 by Bonferroni.

#### A relationship between various characteristics and HGS of subjects categorized by gender

**Fig 1:**
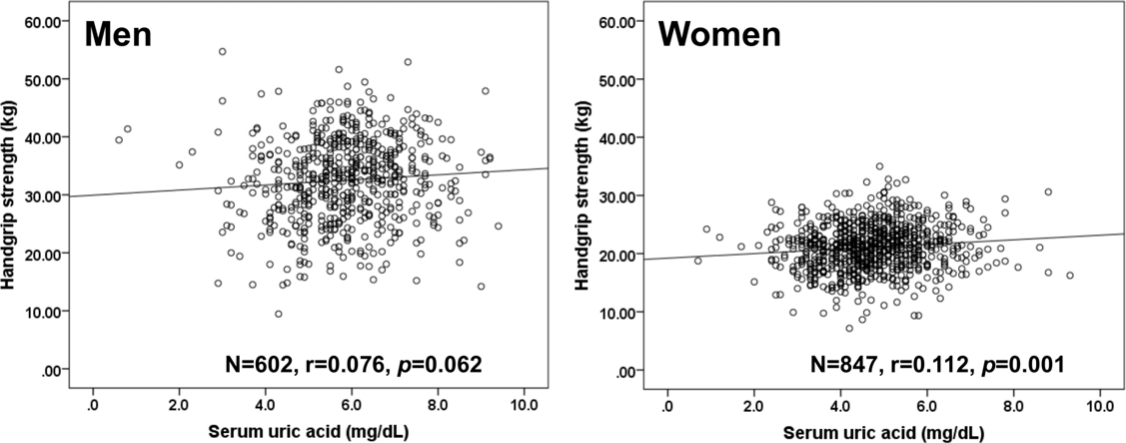
Relationship between serum uric acid (UA) and handgrip strength (HGS) by gender. Solid line, men; dashed line, women. Serum UA levels were significantly correlated with HGS in men (r = 0.138, *p*<0.001), but not in women (r = 0.071, *p* = 0.029). There was a significant interaction between gender and increased serum UA on HGS (F = 5.313, *p* = 0.021).

As shown in Fig 1, serum UA levels were significantly correlated with HGS only in women (r = 0.112, *p* = 0.001), but not in men (r = 0.076, *p* = 062). Table 5 shows the age-adjusted relationship between participant characteristics and HGS of the subjects categorized by gender. In men, BMI, drinking status, LDL-C, and eGFR_CKDEPI_ were significantly correlated with HGS levels and in women, BMI, drinking status, DBP, presence of antidyslipidemic medication, eGFR_CKDEPI_, and serum UA were significantly correlated with HGS.

**Table 5:** Age-adjusted relationship between various characteristics and handgrip strength categorized by gender.

	Handgrip strength	
	Men N = 602	Women N = 847
Characteristic	Partial r (*P*-value*)	Partial r (*P*-value*)
Body mass index	0.097 (0.017)	0.140 (<0.001)
Exercise habits (No = 0, Yes = 1)	0.019 (0.648)	0.015 (0.673)
Smoking status	0.008 (0.851)	-0.023 (0.501)
Drinking Status	0.123 (0.002)	0.097 (0.005)
Systolic blood pressure	0.025 (0.535)	0.062 (0.070)
Diastolic blood pressure	0.077 (0.058)	0.112 (0.001)
Antihypertensive medication (No = 0, Yes = 1)	0.031 (0.453)	0.017 (0.630)
Triglycerides	0.036 (0.384)	0.020 (0.570)
HDL cholesterol	0.068 (0.095)	-0.034 (0.324)
LDL cholesterol	0.144 (<0.001)	0.023 (0.506)
Antidyslipidemic medication (No = 0, Yes = 1)	0.027 (0.512)	0.085 (0.013)
Hemoglobin A 1c	-0.013 (0.756)	0.024 (0.493)
Antidiabetic medication (No = 0, Yes = 1)	-0.043 (0.294)	-0.021 (0.549)
eGFR_CKDEPI_	-0.139 (0.001)	-0.147 (<0.001)
Serum uric acid	0.050 (0.221)	0.157 (<0.001)

r, Pearson’s correlation coefficient.

*Adjusted for age. Data for triglycerides was skewed and log-transformed for analysis.

#### Multiple linear regression model testing the relationship between serum UA and HGS of subjects categorized by gender

To further investigate whether serum UA can explain HGS independent of other confounding factors, multiple linear regression analysis using HGS as an objective variable and various confounding factors as explanatory variables were performed with subjects categorized by gender (Table 6). In men, age, drinking status, HDL-C, LDL-C, and eGFR_CKDEPI_ were independently and significantly associated with HGS, and in women, serum UA as well as age, BMI, drinking status, DBP, and eGFR_CKDEPI_ were independently and significantly associated with HGS

**Table 6:** Multiple linear regression models testing the relationship between serum uric acid and Handgrip strength categorized by gender.

	Handgrip strength			
	Men N = 602 Women N = 847			
	Forced	Backward elimination	Forced	Backward elimination
Characteristic	β (*P*-value*)	β (*P*-value*)	β (*P*-value*)	β (*P*-value*)
Age	-0.590 (<0.001)	-0.619 (<0.001)	-0.505 (<0.001)	-0.541 (<0.001)
Body mass index	0.059 (0.109)	0.062 (0.069)	0.100 (0.003)	0.093 (0.004)
Exercise habits	0.011 (0.741)	——	0.021 (0.474)	——
Smoking status	0.020 (0.546)	——	-0.025 (0.410)	——
Drinking Status	0.110 (0.002)	0.104 (0.002)	0.084 (0.007)	0.081 (0.008)
Systolic blood pressure	-0.048 (0.329)	——	-0.048 (0.322)	——
Diastolic blood pressure	0.066 (0.203)	——	0.119 (0.011)	0.087 (0.005)
Antihypertensive medication	-0.010 (0.779)	——	-0.046 (0.165)	-0.056 (0.086)
Triglycerides	0.025 (0.490)	——	-0.050 (0.153)	——
HDL cholesterol	0.079 (0.039)	0.071 (0.042)	-0.030 (0.390)	——
LDL cholesterol	0.119 (<0.001)	0.124 (<0.001)	0.031 (0.343)	——
Antidyslipidemic medication	0.031 (0.353)	——	0.068 (0.038)	0.055 (0.075)
Hemoglobin A 1c	0.004 (0.920)	——	0.007 (0.845)	——
Antidiabetic medication	-0.028 (0.441)	——	-0.032 (0.372)	——
eGFR_CKDEPI_	-0.146 (<0.001)	-0.142 (<0.001)	-0.116 (0.003)	-0.117 (0.003)
Serum uric acid	-0.019 (0.596)	——	0.073 (0.037)	0.069 (0.044)
R2	0.404 (<0.001)	0.399 (<0.001)	0.270 (<0.001)	0.254 (<0.001)

β, standardized coefficient, R^2^, coefficient of determination.

*Adjusted for all confounding factors. Data for triglycerides was skewed and log-transformed for analysis. (——) did not remain in the final model by multiple linear regression analysis.

#### Age and Multivariate-adjusted HGS of subjects categorized by gender and quartiles of serum UA

Table 7 shows age and multivariate-adjusted HGS of men and women categorized by each quartiles of serum UA. In women, age and multivariate-adjusted HGS was significantly higher in the Quartile-3 and Quartile-4 groups of serum uric acid than in the lower groups.

**Table 7:** Age and Multivariate-adjusted handgrip strength of subjects categorized by gender and quartile of serum uric acid.

	Serum uric acid (mg/dL)				
	Quartile-1	Quartile-2	Quartile-3	Quartile-4		
	0.6–5.1	5.2–5.8	5.9–6.7	6.8–9.4	
**Men N = 602**	**N = 165**	**N = 136**	**N = 161**	**N = 140**	*P* for trend*
Mean handgrip strength (kg)					
Age-adjusted	31.6 (30.7–32.5)	32.6 (31.6–33.6)	33.0 (32.1–33.9) †	32.9 (32.0–33.9) †	0.101
Multivariate-adjusted	32.0 (31.1–32.9)	32.8 (31.8–33.8)	33.0 (32.1–33.9)	32.3 (31.3–33.3)	0.409
	**0.7–4.0**	**4.1–4.7**	**4.8–5.4**	**5.5–9.3**	
**Women N = 847**	**N = 233**	**N = 222**	**N = 198**	**N = 194**	*P* for trend*
Mean handgrip strength (kg)					
Age-adjusted	20.3 (19.9–20.7)	20.5 (20.0–20.9)	22.0 (21.5–22.5) ‡ #	21.7 (21.2–22.2) ‡ #	<0.001
Multivariate-adjusted	20.6 (20.1–21.0)	20.6 (20.2–21.1)	21.9 (21.4–22.4) ‡ #	21.4 (20.9–21.9) † $	<0.001

Multivariate-adjusted for age, body mass index, exercise habits, smoking status, drinking status, systolic blood pressure, diastolic blood pressure, antihypertensive medication, high-density lipoprotein cholesterol, low-density lipoprotein cholesterol, triglycerides, antidyslipidemic medication, hemoglobin A1c, antidiabetic medication, and eGFR_CKDEPI_. Data for triglycerides was skewed and log-transformed for analysis.

*P for trend: ANCOVA.

† *P*<0.05

‡ *P*<0.001 vs. Quartile-1, and

$ *P*<0.05

# *P*<0.001 vs. Quartile-2 by Bonferroni.

## Discussion

This study demonstrated that serum UA levels are positively associated with HGS after adjusting for potential confounders in Japanese adult women aged 60–90 years.

These results suggest that serum uric acid may have a protective role in aging-associated decline in muscle strength in community-dwelling elderly women. To our knowledge, few epidemiology studies have quantified the link between serum uric acid and HGS in community-dwelling Japanese elderly women

Several prospective and cross-sectional studies have found that the link between hyperuricemia and HGS has been reported. Ruggiero et al. [18] demonstrated that participants within the middle serum UA quintile (4.8–5.3 mg/dL) were less disabled in instrumental activities of daily living than those in the extreme serum UA quintiles among 966 elderly persons aged 65 years and older. Among 586 Japanese male employees aged 30 years and older, Huang et al. reported that muscle strength was much lower in persons with hyperuricemia than in those without hyperuricemia and serum UA levels (quartiles) showed an inverted J-shaped curve with HGS {mean and 95% CI: Quartile-1, 41.6 (40.6–42.6) kg; Quartile-2, 42.2 (41.2–43.2) kg; Quartile-3, 41.8 (40.8–42.8) kg; Quartile-4, 40.4 (39.3–41.4) kg; *P* for trend = 0.05) [19]. These results are cross-sectional, and suggest that keeping serum UA at an optimal level may contribute to maintaining skeletal muscle mass. The findings of Wu et al. [13], however, are in contrast to these. From Chinese aged 50–74 years, they also reported that HGS levels increased significantly across serum UA tertiles (26.4 ± 8.5 kg; 30.1 ± 10.5 kg; 35.0 ± 11.4 kg; *P*<0.001), and after adjusting for potential confounders, high serum UA level remained significantly associated with high grip strength (*P* = 0.023). Also, during the 497 InCHIANTI study, for participants aged 76.0 ± 5.4 years, follow-up HGS measurements increased significantly across baseline serum UA tertiles, and after adjusting for potential confounders and analogous baseline strength measures, higher baseline serum UA levels still remained significantly associated with higher follow-up strength measurements [12]. In 7,544 men and women aged 40 years of age and older, increased serum UA was significantly related to sarcopenia status, and participants in the highest group of serum UA level (>8 mg/dL) had 2.0 times the odds of manifesting sarcopenia compared to those in the lowest group (< 6 mg/dL) (*p*< 0.01) after adjusting for the confounders [20]. In our study, the association of serum UA with HGS was observed in women only. These confiicting findings are partly related to methodological differences and to participant characteristics. In addition, as serum UA revels relate with increasing numbers of or special metabolic risk factors, the effect of UA on muscle strength might become negligible. It is very interesting to note a J-shaped association of serum UA levels with CVD events [21] and all-cause mortality [22, 23], implying that both lower and higher serum UA levels lead to a higher risk.

We thought that sex-specific analyses were also required because at all ages, serum UA level and handgrip strength are higher in men than in women. We cannot explain the underlying mechanism that accounts for the gender difference from this study. A partial explanation for this result could be alcohol consumption, which is more likely to be higher in men, the use of antihypertensive drugs such as diuretics, which are known to increase serum UA levels [24], and the influence of sex hormones due to the promotion of excretion of uric acid by estrogen [25]. In our study, the analysis was performed after adjusting for alcohol consumption and antihypertensive medication. Effects of sex hormone require further investigation in the future. In addition, the precise mechanisms that antioxidant effects of uric acid were different between the two genders in our study are not completely understood. UA has been known to be a neuroprotective antioxidant because of its free radical scavenger activity [26, 27], and Llull et al. have demonstrated that UA might lessen greater disability after stroke more in women than in men [28]. Serum UA may exerts more potential antioxidant effects also on the skeletal muscle function in women than in men.

The mechanisms that lead to stronger HGS in individuals with serum UA at an optimal level remains to be clarified. A recent study [29] has shown that oxidative protein damage is independently associated with low HGS among older persons, suggesting that oxidative stress might contribute to the loss of muscle strength and mass. Urate crystals contribute to the inflammatory response through the release of pro-inflammatory mediators [30], and the risk of urate crystal formation/precipitation increases when serum UA level exceeds 6.3 mg/dL [31]. Serum UA may alter the proliferation/migration of nitric oxide (NO) release from human vascular cells that is mediated by the expression of C-reactive protein (CRP) [32]. In addition, serum UA stimulates proliferation of angiotensin II production, and oxidative stress in vascular smooth muscle cells through the tissue renin-angiotensin system [33]. Moreover, serum UA was associated positively with IL-6 (IL-6), CRP and TNF-α, particularly in women [34]. CRP, TNF-α, and IL-6, which are a prominent markers of systemic chronic inflammation, have been significantly associated with poor HGS [35, 36, 37]. The negative impact of high serum UA levels on muscle strength may be largely due to the serum UA-induced pro-oxidant capacity (primarily within the cell) at higher than normal levels [19]. In addition, serum UA reduces endothelial nitric oxide (NO) levels, which increase blood flow to skeletal muscles and enhance glucose uptake, and strongly relate to insulin action [38]. Serum UA, at optimal levels, is a powerful antioxidant and a scavenger of singlet oxygen and radicals [7]. Waring et al. [39] have shown the protective effect of UA on oxidative stress generated during physical activity. Given its powerful antioxidant capacity, serum UA may protect skeletal muscle function from ROS-induced protein oxidative damage.

Several limitations should be considered in this study. First, our cross-sectional study design does not eliminate potential causal relationships between serum UA and muscle function. Second, serum UA categories are based on a single assessment of blood, which may introduce a misclassification bias. Third, we could not eliminate the possible effect of medications for hypertension and dyslipidemia, and hormone replacement treatment on the present findings. Fourth. we could not eliminate the possible effects of underlying diseases (e.g., under nutrition due to various illness and healthy diet and consequently higher serum uric acid levels) on the present findings. Therefore the demographics and referral source may limit generalizability.

## Conclusions

The present study showed that serum UA is strongly associated with muscle function among Japanese community-dwelling elderly women. The underlying mechanism behind this relationship is unclear, but seems to be independent of traditional confounding factors such as age, BMI, smoking status, alcohol consumption, blood pressure, lipids, or renal function. Thus, serum UA levels in elderly women might provide an important marker for the assessment of risk as well as a therapeutic target for the modification of sarcopenia. For healthy community-dwelling elderly women, prospective population-based studies are needed to investigate the mechanisms underlying this association to determine whether intervention, such as effective lifestyle modifications and medications that control serum UA in adults, will improve muscle function.

## References

[1] Ruggiero C, Cherubini A, Ble A, Bos AJ, Maggio M, Dixit VD, et al Uric acid and inflammatory markers. Eur Heart J. 2006; 27: 1174–1181. 16611671 10.1093/eurheartj/ehi879 PMC2668163

[2] Zoccali C, Maio R, Mallamaci F, Sesti G, Perticone F. Uric acid and endothelial dysfunction in essential hypertension. J Am Soc Nephrol. 2006; 17: 1466–1471. 16611716 10.1681/ASN.2005090949

[3] Sundström J, Sullivan L, D'Agostino RB, Levy D, Kannel WB, Vasan RS. Relations of serum uric acid to longitudinal blood pressure tracking and hypertension incidence. Hypertension. 2005; 45: 28–33. 15569852 10.1161/01.HYP.0000150784.92944.9a

[4] Kawamoto R, Tabara Y, Kohara K, Kusunoki T, Abe M, Miki T. Serum uric acid is more strongly associated with impaired fasting glucose in women than in men from a community-dwelling population. PLoS One. 2013; 8: e65886 10.1371/journal.pone.0065886 23785457 PMC3681777

[5] Meisinger C, Koenig W, Baumert J, Döring A. Uric acid levels are associated with all-cause and cardiovascular disease mortality independent of systemic inflammation in men from the general population: the MONICA/KORA cohort study. Arterioscler Thromb Vasc Bio. 2008; 28: 1186–1192. 18356554 10.1161/ATVBAHA.107.160184

[6] Glantzounis GK, Tsimoyiannis EC, Kappas AM, Galaris DA. "Uric acid and oxidative stress". Curr Pharm Des. 2005; 11: 4145–4151. 16375736 10.2174/138161205774913255

[7] Ames BN, Cathcart R, Schwiers E, Hochstein P. Uric acid provides an antioxidant defense in humans against oxidant- and radical-caused aging and cancer: a hypothesis. Proc Natl Acad Sci U S A. 1981; 78: 6858–6862. 6947260 10.1073/pnas.78.11.6858 PMC349151

[8] Kohara K. Sarcopenic obesity in aging population: current status and future directions for research. Endocrine. 2014; 45: 15–25. 10.1007/s12020-013-9992-0 23821364

[9] Kim TN, Choi KM. Sarcopenia: Definition, Epidemiology, and Pathophysiology. J Bone Metab. 2013; 20: 1–10. 10.11005/jbm.2013.20.1.1 24524049 PMC3780834

[10] Fulle S, Protasi F, Di Tano G, Pietrangelo T, Beltramin A, Boncompagni S, et al The contribution of reactive oxygen species to sarcopenia and muscle ageing. Exp Gerontol. 2004; 39: 17–24. 14724060 10.1016/j.exger.2003.09.012

[11] Derbré F, Gratas-Delamarche A, Gómez-Cabrera MC, Viña J. Inactivity-induced oxidative stress: a central role in age-related sarcopenia? Eur J Sport Sci. 2014; 14: S98–108. 10.1080/17461391.2011.654268 24444251

[12] Macchi C, Molino-Lova R, Polcaro P, Guarducci L, Lauretani F, Cecchi F, et al Higher circulating levels of uric acid are prospectively associated with better muscle function in older persons. Mech Ageing. 2008; 129: 522–527. 10.1016/j.mad.2008.04.008 PMC2600487 18534661

[13] Wu Y, Zhang D, Pang Z, Jiang W, Wang S, Tan Q. Association of serum uric acid level with muscle strength and cognitive function among Chinese aged 50–74 years. Geriatr Gerontolol Int. 2013; 13: 672–677. 10.1111/j.1447-0594.2012.00962.x 23170844

[14] Cruz-Jentoft AJ, Baeyens JP, Bauer JM, Boirie Y, Cederholm T, Landi F, et al; European Working Group on Sarcopenia in Older People. Sarcopenia and mortality risk in frail older persons aged 80 years and older: results from ilSIRENTE study. Age Ageing. 2010; 39: 412–423. 10.1093/ageing/afq034 20392703 PMC2886201

[15] Kawamoto R, Tabara Y, Kohara K, Miki T, Kusunoki T, Abe M, et al High-sensitivity C-reactive protein and gamma-glutamyl transferase levels are synergistically associated with metabolic syndrome in community-dwelling persons. Cardiovascular. Diabetology. 2010; 9: 87. 10.1186/1475-2840-9-87 PMC3014885 21143879

[16] Kawamoto R, Tabara Y, Kohara K, Miki T, Kusunoki T, Takayama S, et al Usefulness of combining serum uric acid and high-sensitivity C-reactive protein for risk stratification of patients with metabolic syndrome in community-dwelling women. Endocrine. 2013; 44: 132–139. 10.1007/s12020-013-9912-3 23475511 PMC3726929

[17] Handgrip strength test: topendsports network. http://www.topendsports.com/testing/tests/handgrip.htm. Accessed 1 July 2014.

[18] Ruggiero C, Cherubini A, Guralnik J, Semba RD, Maggio M, Ling SM, et al The interplay between uric acid and antioxidants in relation to physical function in older persons. J Am Geriatr Soc. 2007; 55: 1206–1215. 17661959 10.1111/j.1532-5415.2007.01260.x PMC2669302

[19] Huang C, Niu K, Kobayashi Y, Guan L, Momma H, Cui Y, et al An inverted J-shaped association of serum uric acid with muscle strength among Japanese adult men: a cross-sectional study. BMC Musculoskelet Disord. 2013; 14: 258 10.1186/1471-2474-14-258 24000893 PMC3766665

[20] Beavers KM, Beavers DP, Serra MC, Bowden RG, Wilson RL. Low relative skeletal muscle mass indicative of sarcopenia is associated with elevations in serum uric acid levels: findings from NHANES III. J Nutr Health Aging. 2009; 13, 177–182. 19262948 10.1007/s12603-009-0054-5 PMC12876367

[21] Johnson RJ, Kang DH, Feig D, Kivlighn S, Kanellis J, Watanabe S, et al Is there a pathogenetic role for uric acid in hypertension and cardiovascular and renal disease? Hypertension. 2003; 41: 1183–1190. 12707287 10.1161/01.HYP.0000069700.62727.C5

[22] Hsu SP, Pai MF, Peng YS, Chiang CK, Ho TI, Hung KY. Serum uric acid levels show a ‘J-shaped’ association with all-cause mortality in haemodialysis patients. Nephrol Dial Transplant. 2004; 19: 457–462. 14736974 10.1093/ndt/gfg563

[23] Suliman ME, Johnson RJ, García-López E, Qureshi AR, Molinaei H, Carrero JJ, et al J-shaped mortality relationship for uric acid in CKD. Am J Kidney Dis. 2006; 48: 761–771. 17059995 10.1053/j.ajkd.2006.08.019

[24] Savage PJ, Pressel SL, Curb JD, Schron EB, Applegate WB, Varadhan R, et al Influence of long-term, low-dose, diuretic-based, antihypertensive therapy on glucose, lipid, uric acid, and potassium levels in older men and women with isolated systolic hypertension: The Systolic Hypertension in the Elderly Program. SHEP Cooperative Research Group. Arch Intern Med. 1998; 158: 741–751. 9554680 10.1001/archinte.158.7.741

[25] Gordon T, Kannel WB. Drinking and its relation to smoking, BP, blood lipids, and uric acid. The Framingham study. Arch Intern Med. 1983; 143: 1366–1374. 6870410

[26] Yu ZF, Bruce-Keller AJ, Goodman Y, Mattson MP. Uric acid protects neurons against excitotoxic and metabolic insults in cell culture, and against focal ischemic brain injury in vivo. J Neurosci Res. 1998; 53: 613–625. 9726432 10.1002/(SICI)1097-4547(19980901)53:5<613::AID-JNR11>3.0.CO;2-1

[27] Romanos E, Planas AM, Amaro S, Chamorro A. Uric acid reduces brain damage and improves the benefits of rt-PA in a rat model of thromboembolic stroke. J Cereb Blood Flow Metab. 2007; 27: 14–20. 16596120 10.1038/sj.jcbfm.9600312

[28] Llull L, Laredo C, Renú A, Pérez B, Vila E, Obach V, et al Uric Acid Therapy Improves Clinical Outcome in Women With Acute Ischemic Stroke. Stroke. 2015; 46: 2162–2167. 10.1161/STROKEAHA.115.009960 26159792

[29] Howard C, Ferrucci L, Sun K, Fried LP, Walston J, Varadhan R, et al Oxidative protein damage is associated with poor grip strength among older women living in the community. J Appl Physiol. 2007; 103: 17–20. 17379753 10.1152/japplphysiol.00133.2007 PMC2646087

[30] Choi HK, Mount DB, Reginato AM. Pathogenesis of gout. Ann Intern Med. 2005; 143: 499–516. 16204163 10.7326/0003-4819-143-7-200510040-00009

[31] Richette P, Bardin T. Gout. Lancet. 2010; 375 (9711): 318–328. 10.1016/S0140-6736(09)60883-7 19692116

[32] Kang DH, Park SK, Lee IK, Johnson RJ. Uric acid-induced C-reactive protein expression: implication on cell proliferation and nitric oxide production of human vascular cells. J Am Soc Nephrol. 2005; 16: 3553–3562. 16251237 10.1681/ASN.2005050572

[33] Mazzali M, Hughes J, Kim YG, Jefferson JA, Kang DH, Gordon KL, et al Elevated uric acid increases blood pressure in the rat by a novel crystal-independent mechanism. Hypertension. 2001; 38: 1101–1106. 11711505 10.1161/hy1101.092839

[34] Lyngdoh T, Marques-Vidal P, Paccaud F, Preisig M, Waeber G, Bochud M, et al Elevated serum uric acid is associated with high circulating inflammatory cytokines in the population-based Colaus study. PLoS One. 2011; 6: e19901 10.1371/journal.pone.0019901 21625475 PMC3098830

[35] Cesari M, Penninx BW, Pahor M, Lauretani F, Corsi AM, Rhys Williams G, et al Inflammatory markers and physical performance in older persons: the InCHIANTI study. J. Gerontol. J Gerontol A Biol Sci Med Sci. 2004; 59: 242–248. 10.1093/gerona/59.3.m242 15031308

[36] Brinkley TE, Leng X, Miller ME, Kitzman DW, Pahor M, Berry MJ, et al Chronic inflammation is associated with low physical function in older adults across multiple comorbidities. J Gerontol A Biol Sci Med Sci. 2009; 64: 455–461. 10.1093/gerona/gln038 19196644 PMC2657165

[37] Schaap LA, Pluijm SM, Deeg DJ, Harris TB, Kritchevsky SB, Newman AB, et al; Health ABC Study. Higher inflammatory marker levels in older persons: associations with 5-year change in muscle mass and muscle strength. J Gerontol A Biol Sci Med Sci. 2009; 64: 1183–1189. 10.1093/gerona/glp097 19622801 PMC2759573

[38] Nakagawa T, Tuttle KR, Short RA, Johnson RJ. Hypothesis: fructose-induced hyperuricemia as a causal mechanism for the epidemic of the metabolic syndrome. Nat Clin Pract Nephrol. 2005; 1: 80–86. 16932373 10.1038/ncpneph0019

[39] Waring WS, Convery A, Mishra V, Shenkin A, Webb DJ, Maxwell SR. Uric acid reduces exercise-induced oxidative stress in healthy adults. Clin Sci (Lond). 2003; 105: 425–430. 12801243 10.1042/CS20030149

